# Biological Activity of the *Salvia officinalis* L. (Lamiaceae) Essential Oil on *Varroa destructor* Infested Honeybees

**DOI:** 10.3390/plants7020044

**Published:** 2018-06-06

**Authors:** Leila Bendifallah, Rachida Belguendouz, Latifa Hamoudi, Karim Arab

**Affiliations:** 1 Laboratory of Soft technology, Valorization, Physical-chemistry of biological materials and Biodiversity, Department of Agronomy, Faculty of Sciences, Université M’hamed Bougara, Boumerdes, Avenue de l’indépendance, Boumerdes 35000, Algeria; 2 Laboratory of Aromatic and Medicinal Plants, Biotechnology Department, Faculty of Nature Sciences and Life, University of Blida, Blida 09000, Algeria; belguendouzr@yahoo.com; 3 Laboratoire de Technologie Alimentaire, Faculté des Sciences de l’ingénieur, Université M’Hamed Bougara, Avenue de L’indépendance, Boumerdès 35000, Algérie; Latifa.Hamoudi.1@ulaval.ca; 4 Laboratoire Valorisation et Conservation des Ressources Biologiques, Department of Agronomy, Faculty of Sciences, Université M’hamed Bougara, Boumerdes 35000, Algeria; arabkarim3@gmail.com

**Keywords:** *Apis mellifera intermissa*, bio-acaricide, Algeria

## Abstract

The present work is conducted as part of the development and the valorization of bioactive natural substances from Algerian medicinal and aromatic spontaneous plants, a clean alternative method in biological control. For this purpose, the bio-acaricidal activity of *Salvia officinalis* (sage)essential oil (EO)was evaluated against the *Varroa destructor*, a major threat to the honey bee *Apis mellifera* ssp. *intermissa*. The aerial parts of *S. officinalis* L., 1753 were collected from the Chrea mountainous area in Northern Algeria. They were subjected to hydro distillation by a Clevenger apparatus type to obtain the EO, and screened for bio-acaricidal activity against *Varroa destructor* by the method of strips impregnated with the mixture EO and twin according to three doses. Pre-treatment results revealed infestation rates in the experimental site ranging from 3.76% to 21.22%. This showed the heterogeneity of infestations in hives according to the density of bees. This constituted a difficulty in monitoring the population dynamics of this parasite. After treatment, a difference in the acaricidal effect of Sage essential oil is noticed. It gives a mortality rate of 6.09% by the dose D1: 5%, 2.32% by the dose D2: 15%, and a low mortality rate of 0.9% by the dose D3: 20%. The chemical treatment carried out by Bayvarol gives a result close to that of the essential oil of Sage (9.97%).These results point to the fact that Sage essential oil treatments have a significant effect and good biological activity with regard to harmful species.

## 1. Introduction

In addition to its different products (honey, royal jelly, pollen), the honeybee plays an important role in the pollination of both cultivated and wild plant species. As a result, it actively contributes to the development and preservation of the biodiversity of ecosystems by promoting the sustainability of flowering plants. It is also considered a true bio-indicator of environmental health [1].

However, the bee is confronted with several constraints that limit its development and that can even cause its disappearance. These constraints are related to the environment (climate and the reduction of food resources), chemical agents (intoxication by phytosanitary products), apicultural practices, and biological agents (bacteria, viruses, parasites, predators).

Among the bee diseases, Varroasisis considered one of the most widespread and dangerous diseases. It is caused by the *Varroa destrucor* Anderson and Trueman, 2000, mite, which parasitizes both brood and adult bees, thus causing considerable losses to the bee population. Several factors, such as the sale of queens and swarms and transhumance, have contributed to the spread of this disease worldwide [2,3].

The losses amount to some thousands of hives just for Eastern Europe [4]. In Algeria, the first varroa was reported in June 1981 on *A. mellifera* ssp. *Intermissa* at Umm-Teboul in Alkala (Annaba). Since then, this parasite has spread to the north of the country [5] and caused the considerable loss of hives. Several methods of physical (heat and fumigation), genetic (breed selection), chemical, and biological control show their efficiency on this parasite, including products like Bayvarol, Apiston, and Apivar and plant extracts such as essential oils of *Eucalyptus Radiata* A. Cunn, *Allium sativum* L., *Rosmarinus officinalis* L., and *Origanum glandulosum* Desf. [6].

Currently, there is lot of interest towards traditional medicines and herbal-based treatment all over the world. Therefore, numerous experimental and clinical studies are being undertaken on medicinal plants and there is a need for updating and integrating the findings [7].

Colin [8] has shown that many plant essential oils have an antiparasite effect, act on the behavior and/or the development of certain arthropods, and can sometimes be fatal.

More than 120 components are characterized in the essential oil prepared from aerial parts of *S. officinalis*. The main components of the oil include borneol, camphor, caryophyllene, cineole, elemene, humulene, ledene, pinene, and thujone [9,10,11]. The sage oil has a direct effect on the nervous system [12]. Camphor, thujone, and terpene ketones are considered as the most toxic compounds in *S. officinalis*. These compounds may induce toxic effects. According to Veličković et al. [13], bornyl acetate, camphene, camphor, humulene, limonene, and thujone are the most comment phytochemicals in the leaves. However, it should be considered that, like other herbs, the chemical composition of *S. officinalis* varies depending on the environmental conditions such as climate, water availability, and altitude [14].

In this work, *Salvia officinalis* (Lamiaceae) is chosen for its acaricidal effect on the *Varroa destructor,* parasite of *Apis mellifera* ssp. *Intermissa*, in comparison with Apivar, which is a commercial chemical product frequently used in Algeria. Our goal is to study the acaricidal activity of its essential oil as a biological alternative to the biopesticide usually practiced in Algeria.

## 2. Results

### 2.1. Estimation of the Initial Infestation Rate in Hives by the Varroa Mite Destructor before Treatment 

The estimated global number of bees on which we conducted our study is 85,000 bees (Table 1). The estimated overall parasitic varroa number is 9801, with an infestation rate of 11.53% judged to be higher than the tolerance threshold established by Robaux [15], which is 5%. This is a strong indicator that this apiary deserves support and immediate treatment.

### 2.2. Estimation of the Infestation Rate in the Different Hives after Treatment with the Essential Oil of Sage Salvia officinalis 

We note that the rate of varroa infection estimated after treatment with the essential oil of sage is 6930 individuals living at the expense of 66,250 bees (Table 2). This number is the equivalent of an infestation rate of 10.46%, judged to be above the tolerance threshold established by Colin [8], which is 5%. This result is directly related to the total number of bees in the apiary, which shows that dense colonies prevent the parasite from overgrowth.

The highest infection rate is that of the control with 26.4%, followed by that of the batch treated with the essential oil and then that treated with the chemical (Bayvarol). This result shows the toxic effect of the oil on the parasite (10.46%), which is more important compared to that of the chemical evaluated at 9.97%.

#### 2.2.1. Comparison between the Effect of the Different Doses of EO in the Batches 01, 02, 03, Treated with the Chemical Treatment

**Figure 1:**
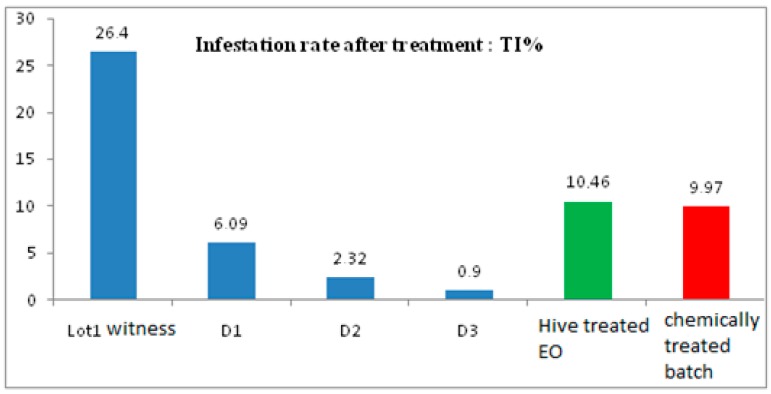
Infestation rate after treatment. EO: Essential Oil.

The comparison between the effect of the different doses of EO in the batches 01, 02, 03, treated with the chemical treatment, is noted in Figure 1.

The essential oil of sage has a toxic effect on varroa, and it is higher (10.46%) than that of chemical treatment (9.97%).

#### 2.2.2. Statistical Analysis of the Results: Variance Analysis

● Number of varroa in the apiary (10 hives) before treatment

**Figure 2:**
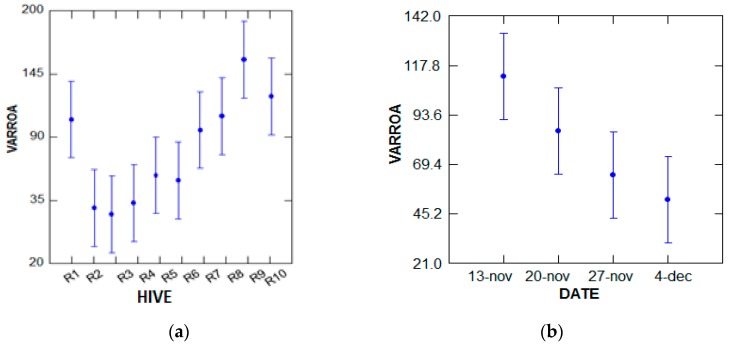
The variance analysis of the number of varroa that died naturally. (**a**) Hive; (**b**) Date.

According to the analysis of the variance performed with the GLM test, the number of varroa that diedvaries marginally (*p* = 0.093, *p* < 5%), depending on the number of bees in the hives, and does not depend on the treatment period. The most populated hives are 10 and 9 during the period of 13 November (Figure 2).

● The number of varroa in the apiary after treatment

**Figure 3:**
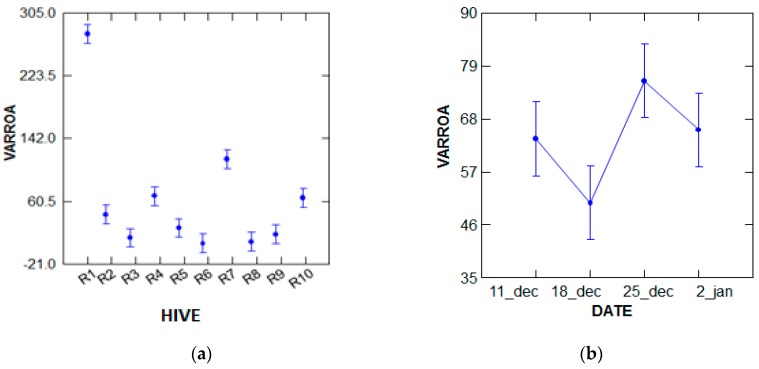
Analysis of the variance of the number of dead varroa by treatment. (**a**) Hive; (**b**) Date.

According to the variance analysis performed with the GLM test, the number of varroa that died varies in a highly significant manner (*p* = 0.000, *p* < 5%), depending on the number of bees in the hives, and does not depend on the number of treatment period. The most populated hives are 01 and 07 during the period of 25 December (Figure 3).

● Global analysis (all factors: date, hive, treatment)

**Figure 4:**
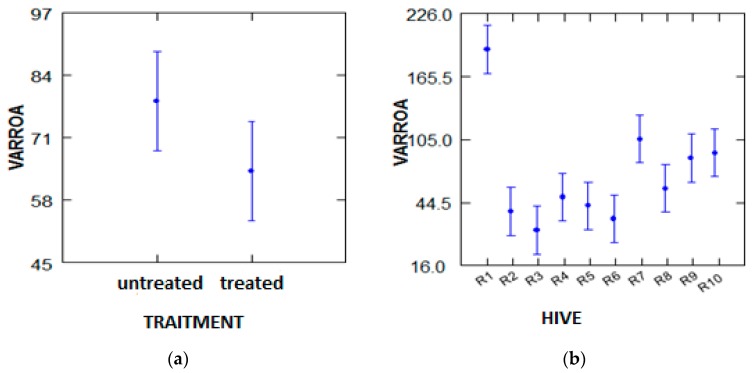
Analysis of the variance of the global number of dead varroa according to the hive factor and treatment. (**a**) Traitment; (**b**) Hive.

According to the hive factor (colonies) and treatment:

According to the variance analysis performed with the GLM test, the number of fallen varroa varies in a highly significant manner (*p* = 0.000, *p* < 5%) depending on the number of bees in the hives, without considering whether they are treated or not treated (Figure 4).

**Figure 5:**
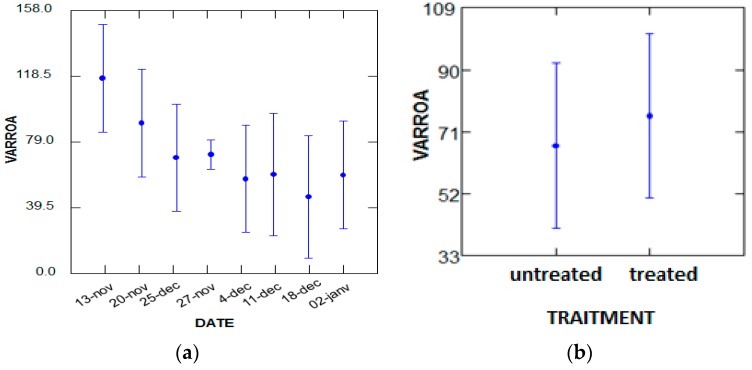
Analysis of the variance of the global number of dead varroa by factor period and treatment. (**a**) Date; (**b**) Traitment.

Depending on the period and treatment factor:

According to the variance analysis performed with the GLM test, the overall number of dead varroa did not depend on the treatment or the treatment period (Figure 5).

## 3. Discussion

Varroasis is a major problem and worrying when considering apiaries, and it has a proliferative capacity, thus causing the annihilation of the colonies of bees. To fight this enemy bee, beekeepers are oriented towards a non-pharmacopoeia reasoned with varroacids, giving birth to cases of resistance accentuated by quantities of residues which upset the products of the hive and human health. The orientation towards natural means such as essential oils of plants aromatics offers a valid solution because their presence is acceptable in the environment of the hive.

Currently, in Europe, several products are applied, and the most used are based on Fluvalinate and Amitraz. It should be noted that no treatment is shown to be 100% effective. Several studies have been conducted, reporting adverse effects of several acaricides on the health of bee colonies [16]. According to Pettis et al. [17], acaricide exposure enhances the susceptibility of bees to diseases and increases bee mortality from these diseases. Varroa mite management requires that acaricide treatments be varroa mite-selective, fatal to varroa mites at doses that are harmless to bees, and leave no or minimal residues in honey and wax [18].

Plants are capable of producing a wide variety of natural substances. In fact, in addition to the classic primary metabolites (carbohydrates, proteins, lipids, nucleic acids), they synthesize and accumulate perpetually secondary metabolites whose physiological function is not always obvious but which represent an immense source of molecules that can be exploited in various fields, among others, phytoprotection [19].

Currently, essential oils are beginning to generate much interest as a potential source of bioactive natural molecules. These products are being studied for their possible use as an alternative to insecticides, acaricides, bactericides, nematicides, and fungicides [20].

In this work, we study the acaricidal activity of *Salvia officinalis* essential oil, which has not been studied so far and comes from the Algiers region, extracted from the leaves of the plant by the hydro-distillation method. The latter allowed us to recover an essential oil yield of 0.56%, which is very low and can be explained by the influence of the region (origin), the sampling period, the climatic conditions, and the method of the extraction.

Our results from the anti-mite treatment reveal an effect of the acaricidal activity of the essential oil of *Salvia officinalis* L. on the parasitic *Varroa destructor* of the honey bee *Apis mellifera intermissa*. This acaricidal activity varies with the dose and the period of exposure to treatment. After treatment, we found that the mortality rate presents a better result when using the dose D1: 5% and that corresponds to 21.07%. This result is weak compared to that of Ghomari et al. [21], who obtained 48.7.20% of the essential oil of *Origanum vulgare*, higher than that obtained by Apivar (3.13%), and much better than that of *Thymus vulgaris,* described as being disappointing according to Giovenazzol et al. [22]. Moussaoui et al. [23] have shown that the toxicity of the *Eucalyptus* bioproduct occurs early.

The bee is an excellent biological indicator. It signals the state of health of the environment in which it lives. It detects the presence of phytosanitary substances, pollutants such as heavy metals, and radionuclides. It also ensures biodiversity through its role as a pollinator. The bee deserves to be protected.

## 4. Materials and Methods

### 4.1. Plant Material

**Figure 6:**
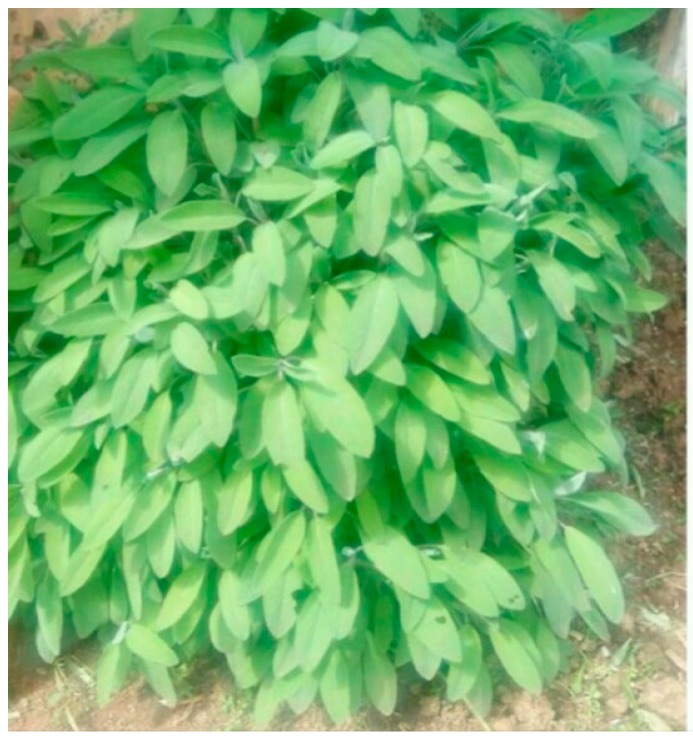
*Salvia officinalis* collected.

The plant used in this study is the spontaneous sage *Salvia officinalis* (Figure 6). In our study, 30-cm apical branches were collected from Chrea, a mountainous area named Tell Atlas, near Blida, Northern Algeria (Latitude: 36°25′32′′ N, Longitude: 2°52′36′′ E, Altitude 946m), just before the appearance of the first floral bud in March 2016.This geographical location offers the plant a typically Mediterranean climate.

They were dried at room temperature and stored in paper bags according of the method of Branislava et al. [24]. The identification of this plant was confirmed according to the general herbarium package available at the High National School of Agronomy of El-Harrach (Algiers).

### 4.2. Animal Material

The *V. jacobsoni* O., 1904 (Arthropod: Dermanissidae or Varroidae) parasite of the domestic honeybee *A. mellifera intermissa* was discovered for the first time on the island Indonesian archipelago on *Apis cerana* (*A. indica*) by the entomologist Jacobson, but his study and description was made by Oudemans in 1904.

This parasite was identified according to the observations of the entomologist teachers at the High National School of Agronomy of El-Harrach—Algiers and Blida University.

**Figure 7:**
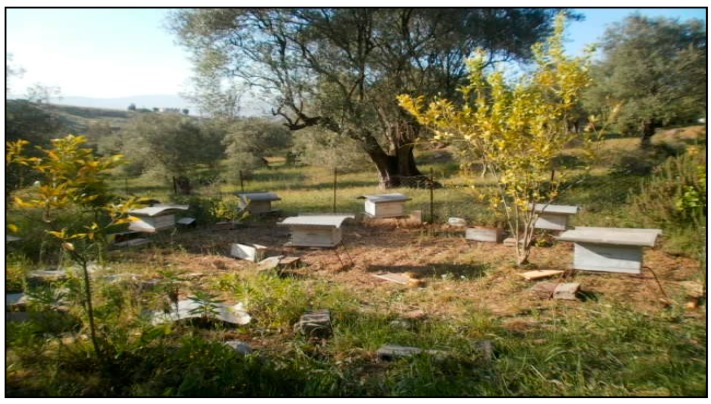
Location of the experimental apiary.

Our study was conducted at the Experimental Station of the Department of Biotechnology, Faculty of Natural Sciences and Life, Blida University I. The apiary has ten hives installed in an orchard consisting of orange trees surrounded by Eucalyptus trees and those of Casuarina (Figure 7).

### 4.3. Method of Extraction of Essential Oil HS from Sage

The freshly harvested plant material was dried with a dryer at a temperature of 37° for 12 h. The aerial parts were cut into small pieces and weighed using a precision scale.

The EO was extracted by the Hydro-distillation method according to the standard procedure reported in the Sixth edition of the European Pharmacopoeia [25], using a Clevenger Type apparatus. This method involves directly immersing the plant material to be treated in a still of distilled water, which is then brought to a boil. The heterogeneous vapors are condensed on a cold surface and the essential oil separates by the difference in density [26]. After 3 h of boiling, the emerged oil is recovered in Eppendorf’s. Distillation is repeated several times with 40g samples.

The yields of essential oils were expressed relative to the dry matter, according to the following formula:

R% = (V/M) × 100

R: percentage of the essential oil.
V: volume obtained in essential oil.
M: weight of the dry material (g).
Preparation of the dilutions
Preparation of 03 dilutions of essential oils to be tested by diluting:
D1: 0.5 mL of essential oils in 100 mL of twin.
D2: 1.5 mL of essential oils in 100 mL of twin.
D3: 02 mL of essential oils in 100 mL of twin.

Then, we prepared strips of blotting paper 18cm long and 5cm wide, each impregnated with 5mL of the different dilutions (D1, D2, D3).

**Figure 8:**
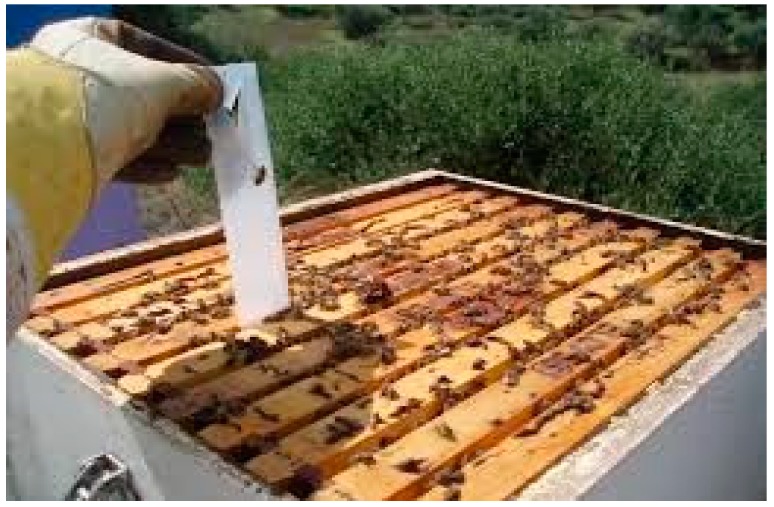
Treatment of colonies with Bayvarol.

For chemical treatment with Bayvarol, we used two strips per hive that were placed vertically between the frames (Figure 8).

### 4.4. Method for Estimating the Initial Infestation Rate of Different Hives

**Figure 9:**
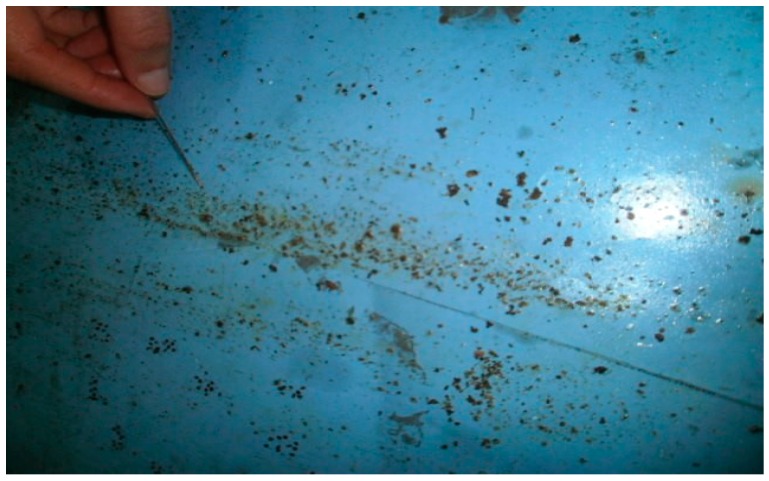
Counting varroa.

We applied one of the biological methods “laying nappies”, using diapers on greased leaves placed on the floor of 10 hives (Figure 9).

The number of varroa was counted weekly for one month before treatment and one month after treatment.

We thus estimated, by a simple division, the mortality.

The daily mortality estimate was made by dividing the total number of Varroa by 29 days; this value was multiplied by 90 days (the maximum life span of Varroa females). This allowed us to obtain the approximate number of existing Varroa in in the colony [26].

### 4.5. Method for Estimating the Number of Bees in a Colony

It was easy for us to estimate the number of bees in our hives, because a Langstroth frame contains 250 grams of bees and the average weight of a bee is estimated at 0.1 g, so a frame would have 2500 bees (250/0.1 = 2500) [27].

### 4.6. Method for Calculating Colony Infestation Rate

After estimating the number of Varroa and bees in a colony, the infestation rate of this colony was estimated as follows:

T · I = C/P

C: corresponds to the number of varroa estimated in a colony.
P: corresponds to the number of bees estimated in a colony [15].

### 4.7. Method for Estimating the Infestation Rate of Different Hives after Treatment

We applied the same method as previously used.

### 4.8. Method for Calculating Colony Infestation Rate

After estimating the number of Varroa that fell after the application of the treatment in the colony, the infestation rate of the colony was evaluated as follows:

TF% = C − M/P

C: corresponds to the number of varroa estimated in the colony before treatment.
M: corresponds to the number of varroa that fell after the treatment.
P: corresponds to the number of bees estimated in a colony [15].

### 4.9. Method for Studying the Phytotoxic Activity of Sage

The essential oil of sage was tested for its phytotoxic activity, at different doses, to obtain the resistance of *Apis mellifera* bees.

In glass jars, five individuals of *A. mellifera* bees were placed on absorbent paper; each beaker contained one dose of sage essential oil (5%, 15%, and 20%) and was covered with a pierced tissue to ensure the bees’ breathing.

We found that the essential oil of sage is active in a toxic way against bees, as follows:

The essential oil of a 5% dose: more than 30 min to notice the beginning of the death of some bees.
The essential oil of a 15% dose: 14.34 min to notice the death of almost all the bees.
The essential oil of a 20% dose: 6.25 min to notice the death of all the bees.

## 5. Conclusions

A preliminary diagnosis can be made after opening brood cells and observations of immature and adult mites present in them or by the biological method “laying nappies”, which reveals, in our study, an initial infestation rate of 11.53%. This rate is close to the range 10 and 20% according to Robaux [15], which means that the colony is strongly affected and requires treatment. This treatment can be done at the level of the hive with various chemicals not without danger since it destroys the mites with negative effects on: the bee, the frames and supports, and the honey. But our study is part of the simple and economical biological method to treat Varroa on the one hand, and collect and observe hive debris and mites for scientific purposes, on the other hand. Itis the method of fumigation treatment using *Salvia officinalis*. It is clear that treatment with Chrea sage reduced the final infestation rate to 10.46, and for the untreated lot, it was 26.4%. Also, statistical analyzes showed that sage essential oil treatments have a significant effect. However, the weakness of the effectiveness of the treatment has its origin in the presence of the capped broods which “protect” the varroa inside the alveoli and thus prevent the penetration of the smoke. In other words, the varroa attached to the lower part of the body of the larva escape, unfortunately, the effects of treatment. Thus, it becomes imperative in our view for beekeepers to ensure the state of the hive before the period of the slope of the eggs to avoid any contamination.

We conclude that the effectiveness of essential oils is related to the plant species, the dose used, and the duration of exposure.

## Figures and Tables

**Table 1:** Estimation of the initial infestation rate (before treatment) in the different hives and lots.

Hive Number	Number of VARROA DIED for 01 Months “A”	Mean Mortality/d B = A/29	Estimated Varroa Population C = B × 90	Estimated Population Bees: P	Initial Infestation Rate T_I_%
**Hive1**	422	14.55	1309	6250	20.94
**Hive 2**	114	3.93	354	8750	4.04
**Witness batch1**	536	18.48	1663	15,000	11.087
**Hive 3**	91	3.13	282	7500	3.76
**Hive 4**	131	4.51	406	8750	4.64
**Batch2**	222	7.64	688	16,250	4.23
**Hive 5**	229	7.87	708	8125	8.71
**Hive 6**	209	7.2	648	11,250	5.76
**Batch3**	438	15.07	1356	19,375	6.99
**Hive 7**	385	13.27	1194	5625	21.22
**Hive 8**	433	14.93	1344	10,000	13.44
**Batch4**	818	28.2	2538	15,625	16.24
**Hives treated per essential oil**	2014	69.39	6245	66,250	9.43
**Hive 9**	644	22.2	1998	10,625	18.8
**Hive 10**	502	17.31	1558	8125	19.18
**Batch5**	1146	39.51	3556	18,750	18.96
**Batch chemically treated**	1146	39.51	3556	18,750	18.97
**global apiary (10 hives)**	**3160**	**108.9**	**9801**	**85,000**	**11.53**

**Table 2:** Estimation of the infestation rate in different hives and batches after inhalation treatment with sage essential oil.

Hive	Infestation Rate after tr: TI%
**Witness batch1**	26.4
**Batch2**	6.09
**Batch3**	2.32
**Batch4**	0.9
**Hives treated/Essential oil**	10.46
**Chemically treated batch**	9.97
